# Moving past the challenges and misconceptions in urban adaptation research

**DOI:** 10.1002/ece3.9552

**Published:** 2022-11-21

**Authors:** Kristin M. Winchell, Kevin J. Aviles‐Rodriguez, Elizabeth J. Carlen, Lindsay S. Miles, Anne Charmantier, Luis F. De León, Kiyoko M. Gotanda, L. Ruth Rivkin, Marta Szulkin, Brian C. Verrelli

**Affiliations:** ^1^ Department of Biology New York University New York NY USA; ^2^ Department of Biology Washington University in St. Louis St. Louis Missouri USA; ^3^ Department of Biology University of Massachusetts Boston Boston Massachusetts USA; ^4^ Department of Biology Fordham University Bronx New York USA; ^5^ Living Earth Collaborative Washington University in St. Louis St. Louis Missouri USA; ^6^ Center for Biological Data Science Virginia Commonwealth University Richmond Virginia USA; ^7^ Centre d'Ecologie Fonctionnelle et Evolutive Université de Montpellier, CNRS, EPHE, IRD Montpellier France; ^8^ Department of Biology Université de Sherbrooke Sherbrooke Quebec Canada; ^9^ Department of Biological Sciences Brock University St. Catharine's Ontario Canada; ^10^ Department of Ecology and Evolutionary Biology University of Toronto Toronto Ontario Canada; ^11^ Department of Biology University of Toronto Mississauga Mississauga Ontario Canada; ^12^ Centre for Urban Environments University of Toronto Mississauga Mississauga Ontario Canada; ^13^ Centre of New Technologies University of Warsaw Warsaw Poland

**Keywords:** anthropogenic, evolutionary ecology, interdisciplinary approaches, natural history, natural selection, urbanization

## Abstract

Although the field of urban evolutionary ecology has recently expanded, much progress has been made in identifying adaptations that arise as a result of selective pressures within these unique environments. However, as studies within urban environments have rapidly increased, researchers have recognized that there are challenges and opportunities in characterizing urban adaptation. Some of these challenges are a consequence of increased direct and indirect human influence, which compounds long‐recognized issues with research on adaptive evolution more generally. In this perspective, we discuss several common research challenges to urban adaptation related to (1) methodological approaches, (2) trait–environment relationships and the natural history of organisms, (3) agents and targets of selection, and (4) habitat heterogeneity. Ignoring these challenges may lead to misconceptions and further impede our ability to draw conclusions regarding evolutionary and ecological processes in urban environments. Our goal is to first shed light on the conceptual challenges of conducting urban adaptation research to help avoid the propagation of these misconceptions. We further summarize potential strategies to move forward productively to construct a more comprehensive picture of urban adaptation, and discuss how urban environments also offer unique opportunities and applications for adaptation research.

## Graphical abstract

In this perspective, we highlight four key challenges to conducting research on adaptive evolution in urban environments. Although these challenges are not unique to urban environments, the increased human dimension can exacerbate these challenges or introduce new applications to adaptation research. Failure to appreciate these challenges can introduce biases and misconceptions, constraining the potential of urban adaptation research to shed light on evolutionary processes generally.

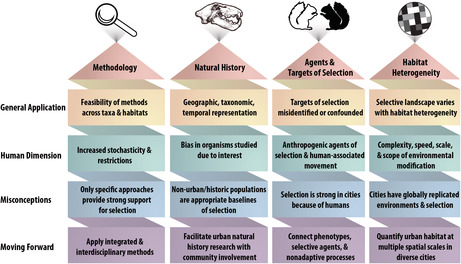

Cities are dramatically different in many dimensions from the non‐urban environments they replace, including structure, species composition, and climate, yet like non‐urban environments, they still host a diverse suite of organisms that interact with each other and the abiotic and biotic environment (Szulkin, Munshi‐South, & Charmantier, 2020). Cities have unique characteristics compared to even other anthropogenic landscapes and are typically characterized by constructed materials, warmer temperatures than the surrounding non‐urban environment, and dense human populations, although there are also green spaces, such as parks and gardens, and landscape features such as rivers and lakes (Johnson & Munshi‐South, 2017; Szulkin, Garroway, et al., 2020; Venter et al., 2016). Some species are filtered out of the urban ecosystem whereas others are able to persist (McDonnell & Hahs, 2015; McKinney, 2002), leading to a range of interacting ecological and evolutionary responses (Alberti, 2015; Alberti et al., 2020). In urban ecosystems, the interaction of human society (e.g., cultural, social, economic, political, and technological) with nature generates complex socio‐eco‐evolutionary dynamics across heterogeneous and novel landscapes (Alberti, 2015; Des Roches et al., 2021; McPhearson et al., 2016; Pincetl, 2015; Rivkin et al., 2019; Schell et al., 2020). We are only beginning to understand how the increased frequency of direct and indirect human influences impacts eco‐evolutionary dynamics as well as the ability of researchers to study them (Miles et al., 2021).

Accumulating evidence to evaluate adaptation—an evolutionary response to natural selection—is challenging in any environment. Difficulties in identifying adaptive evolution stem from the complexity of the processes facilitating or impeding responses: mutation, gene flow, genetic drift, and natural selection (Kawecki & Ebert, 2004). These processes are dependent on life history, habitat use, and movement throughout the landscape, with variable influence and interaction across spatial and temporal scales (Hoban et al., 2016; Levin, 1992; Olson‐Manning et al., 2012). Challenges to adaptation research in general have been extensively treated elsewhere (e.g., Blanquart et al., 2013; Endler, 1986; Kawecki & Ebert, 2004). In addition, a number of reviews of urban evolutionary ecology have provided excellent syntheses on eco‐evolutionary processes, including adaptation, in urban ecosystems (Alberti, 2015; Des Roches et al., 2021; Diamond et al., 2022; Diamond & Martin, 2021; Donihue & Lambert, 2015; Johnson & Munshi‐South, 2017; Lambert et al., 2021; McDonnell & Hahs, 2015; Miles et al., 2019; Rivkin et al., 2019; Szulkin, Munshi‐South, & Charmantier, 2020). However, what is missing from this discourse is an overall reflection on how conducting adaptation research is challenged by the human dimension of socio‐cultural and ecological influence in urban ecosystems.

The aim of this perspective is to highlight challenges in urban adaptation research, and outline strategies to move forward, including the discussion of opportunities generated by this fascinating field of research. Our unique perspective brings these ideas together in a framework that provides both conceptual and practical advice with the goal of providing guidance to researchers, especially those in early career positions, regarding the pitfalls that can hinder success in urban adaptation research. In not considering these challenges, urban researchers may unintentionally propagate misconceptions – inaccurate conclusions as a result of faulty information – regarding adaptation. These misconceptions can include the commonality, nature, and strength of adaptive responses, and can influence expectations based on non‐urban ecosystems or suggest methods that may not be applicable across diverse habitats and taxa.

We explore four challenges commonly encountered when conducting adaptation research and which can be further compounded by the human dimension in urban environments: (1) methodological approaches, (2) trait–environment relationships and natural history, (3) agents and targets of natural selection, and (4) habitat heterogeneity. For each challenge, we employ a four‐point framework to bring together ideas from the fields of urban ecology and evolutionary biology, adaptation research more generally, and urban adaptation research specifically. We first note how each challenge applies to adaptation research in any ecosystem, and explore how the human dimension in urban areas can play a specific role in adaptation. We then summarize the misconceptions that can arise and potential ways to move forward using examples from the urban evolutionary ecology literature. We conclude by emphasizing the opportunities and applications of conducting research on urban adaptation. We recognize that many of these ideas have been addressed throughout the literature and that they may not *all* be novel to *every* urban environment. In the coming decades with predicted novel research directions in urban evolutionary ecology incorporating technology, sustainability, climate change, and socio‐political considerations (Verrelli et al., 2022), we see our perspective as providing a valuable primer to those entering this burgeoning field from many different disciplines.

## 1 CHALLENGE 1: METHODOLOGICAL APPROACHES

### 1.1 General application

Studies of adaptation have historically relied on a mix of observational and experimental methods. Adaptation research often focuses on divergent habitats, although clines across environmental transitions have also been instrumental in studying local adaptation (Endler, 1986; Hereford, 2009; Kawecki & Ebert, 2004). Yet it can be difficult to define the boundaries of habitats and populations in heterogeneous landscapes (see Challenge 4) and environmental variation may present as mosaics rather than gradients. Certain organisms may be more tractable for the quantification of natural selection because of their reproductive cycle, demography, generation time, and geography, which may bias the organisms we choose to study. In particular, approaches requiring the movement of organisms between habitats, such as reciprocal transplantation, are not feasible for all organisms, can be prohibitively expensive and time‐consuming, may require unattainably large numbers of replicates to obtain sufficient statistical power, can facilitate the spread of diseases and parasites, and may be impossible for ethical or legal reasons (Blanquart et al., 2013; Cunningham, 1996; Johnson et al., 2021; Kawecki & Ebert, 2004). Common approaches for adaptation research, such as mark‐recapture and long‐term monitoring, which have been crucial in disentangling the temporal dynamics of adaptive evolution (e.g., Grant & Grant, 2014), may be compromised by external factors such as natural disasters and logistics of carrying out such projects (e.g., funding and researcher continuity). Genomic approaches to identifying local adaptation are becoming increasingly common and may be valuable complements to field research methods, yet genomic approaches come with their own methodological limitations as well (Hoban et al., 2016; Perrier et al., 2020). Lastly, interpersonal interactions between researchers and local community members in any environment can be friendly and educational—offering opportunities for broader impacts of research activities—but can also pose safety risks for researchers (Demery & Pipkin, 2021).

### 1.2 Human dimension

Some methods that may be relatively easy to employ in non‐urban settings may be untenable in urban environments (or vice versa). Urban adaptation can be influenced by factors related to increased human activity that are difficult to control using traditional manipulative experiments or may be difficult to predict. Direct and indirect human interactions with wildlife can shape behavioral responses and adaptations (e.g., pedestrian behavior, Bateman & Fleming, 2014) and human activities can drastically transform urban environments even on short timescales (see Challenge 3). Rapid or unanticipated anthropogenic modifications in cities limit the establishment and success of studies that involve repeated sampling and long‐term monitoring (McPhearson et al., 2016). The mosaic of private and public lands in urban environments intersecting with human and wildlife activity adds additional complexity to the methods that can be employed to conduct urban adaptation research. For example, mark‐recapture methods to estimate selection gradients can be challenging because marked individuals can move into inaccessible anthropogenic spaces that dominate urban landscapes, such as restricted‐access private property (e.g., backyards or inside homes). Similarly, a random sample of the environment for population genomic analyses could be hampered by private property access in non‐random ways across the urban landscape. Some methods, such as reciprocal transplants, may also unintentionally facilitate human–wildlife conflict (Kansky et al., 2016; Schell et al., 2020; Treves et al., 2006), disease transmission between urban wildlife and domesticated animals and humans (Bradley & Altizer, 2007; Brearley et al., 2013), and biological invasion (Borden & Flory, 2021; Hufbauer et al., 2012). Community members tend to be more concerned and vocal about these potential threats when they occur near their homes (Dickman et al., 2014; Drake et al., 2020). Additionally, urban areas are characterized by a higher human density, which increases interactions between researchers and the public and law enforcement, both positive and negative, and can be problematic when urban sites are repeatedly accessed (Des Roches et al., 2021; Dyson et al., 2019).

### 1.3 Misconceptions

A misconception perpetuated by methodological challenges to urban adaptation research is that only specific approaches, such as reciprocal transplants, provide strong support for local adaptation (e.g., Diamond et al., 2022; Donihue & Lambert, 2015; Lambert et al., 2021). Although common garden and reciprocal transplant studies are informative for evaluating evidence of local adaptation in some taxa, such as invertebrates or plants (Chick et al., 2020; Diamond et al., 2022; Gorton et al., 2018; Tüzün & Stoks, 2020; Yakub & Tiffin, 2017; Yilmaz et al., 2020), they are not informative or feasible for all taxa and informativeness may depend on gene flow or other natural history characteristics (see Challenges 2 and 3). Advocating broadly for “gold standard” methods might lead to an overrepresentation in urban adaptation research of organisms, microhabitats, or geographic regions most amenable to these approaches. Extrapolating findings based on a restricted set of methods or taxa could lead to incorrect conclusions regarding the generalizability and prevalence of urban adaptive responses.

### 1.4 Moving forward

**FIGURE 1:**
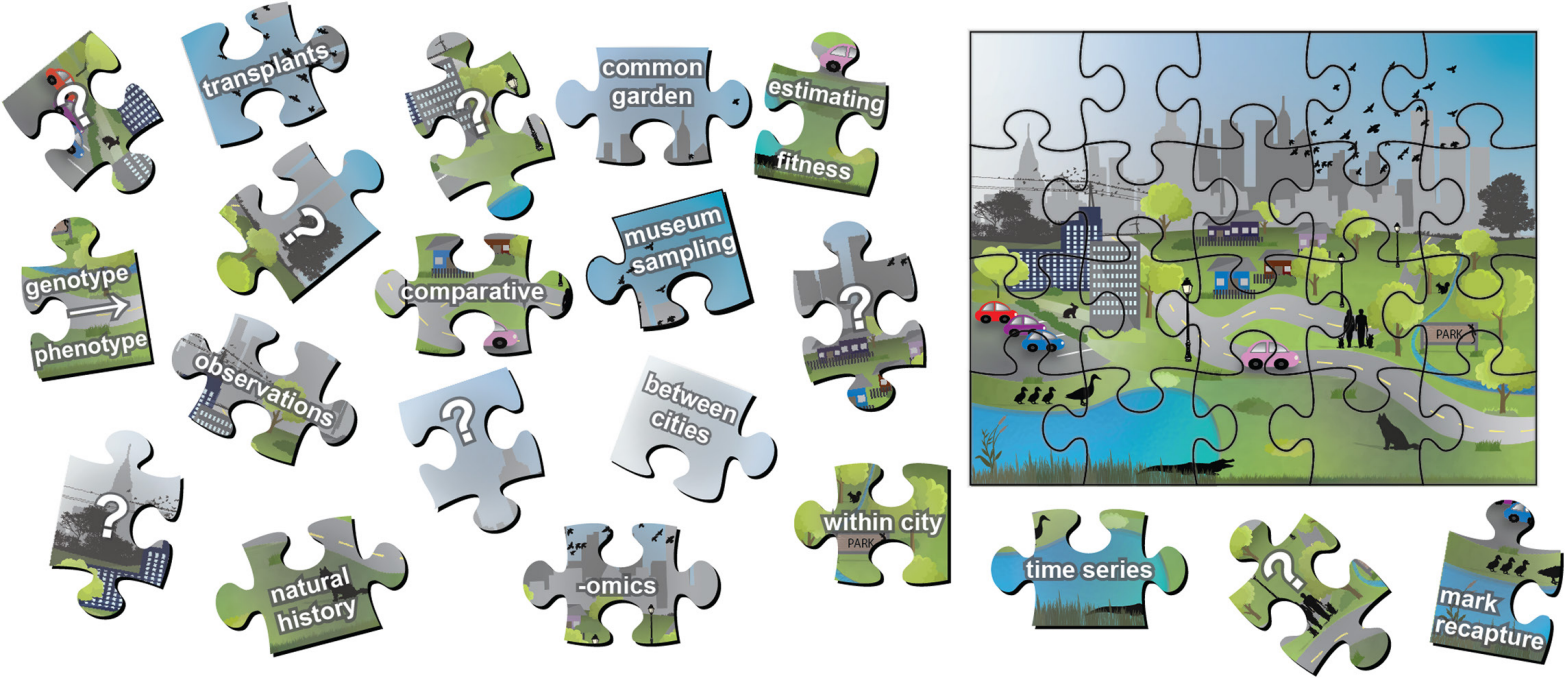
Completing the puzzle. Just as the picture on a puzzle cannot be fully understood from a single piece, no single method can tell us all we need to learn about adaptation in any environment, including urban environments, and each piece can tell us something uniquely important. When pieced together, we obtain a more comprehensive picture of adaptation. Employing complementary approaches that account for both taxonomic and environmental variation provides different pieces of evidence to better understand evolutionary processes and patterns in general and may help move adaptation research in urban ecosystems forward productively.

To address the methodological challenges associated with human presence and activity in urban landscapes, research efforts that employ complementary and innovative methods will provide different pieces of the adaptation puzzle (Figure 1). As in non‐urban environments, multifaceted approaches will be most robust for detecting and characterizing local adaptation (Barrett & Hoekstra, 2011; Kawecki & Ebert, 2004). As a result of increased human interactions in urban areas, collaborations among diverse disciplines can become more commonplace and bring new technology and methodology into urban adaptation research (McPhearson et al., 2016). Interdisciplinary approaches may be particularly valuable in urban ecosystems, where both empirical and applied science involve human activities and have the potential to promote human well‐being (McPhearson et al., 2016). Inclusion of local communities in urban and non‐urban systems alike can improve the success of methodological approaches via the incorporation of local knowledge (Camacho et al., 2021; Uprety et al., 2012) and will help improve researcher outcomes in terms of safety, access, and study continuity (e.g., continued or repeated access, reduced vandalism). There are several examples where integrated approaches have been used to build a more comprehensive picture of urban adaptation: research on *Anolis* lizards has incorporated behavioral, phenotypic, experimental, and genomic analyses to understand adaptation to thermal and structural habitats (Avilés‐Rodríguez & Kolbe, 2019; Campbell‐Staton et al., 2020; Winchell et al., 2016); work on white clover (*Trifolium repens*) has involved the global community in sampling efforts complemented with experimental, phenotypic, and whole genome sequencing analyses to test for parallelism (Santangelo et al., 2022; Santangelo, Thompson, et al., 2020; Thompson et al., 2016); research on Galapagos finches (*Geospiza* spp.) has employed morphometrics and behavioral approaches to understand how access to human foods alter historical patterns of diet‐based selection on beak shape (De León et al., 2011, 2018); and a combination of reciprocal transplants, phenotypic variation, and mate choice experiments in Tungara frogs (*Engystomops pustulosus*) has revealed adaptive sexual selection (Halfwerk et al., 2019).

## 2 CHALLENGE 2: TRAIT–ENVIRONMENT RELATIONSHIPS AND THE NATURAL HISTORY OF ORGANISMS

### 2.1 General application

Understanding the natural history of an organism, including how it interacts with the environment, provides the foundation for conducting hypothesis‐driven adaptation research (Greene, 1986; Tewksbury et al., 2014). Conducting research on trait–environment relationships and natural history is challenging because it requires time‐consuming and detailed studies of how organisms utilize environmental spaces, which may differ on spatial and temporal scales and may be difficult to accomplish for cryptic or elusive taxa (Morris, 1987). Adaptation research can benefit from museum collections to understand historical and contemporary selective landscapes (Shultz et al., 2020; Wandeler et al., 2007), although geographic, taxonomic, and temporal bias in specimen collection limit our ability to universally rely on this resource (Vawda, 2019). One benefit of museum specimens is in potential genomic analyses, which can be challenged by obtaining high‐quality DNA yet new methods applied to ancient DNA (e.g., Castañeda‐Rico et al., 2020) are promising and open up new opportunities for exploring temporal trends. In addition, plasticity can modify trait–environment relationships on local scales (Lajoie & Vellend, 2015, 2018), and thus, can make it difficult to make generalizations about trait–environment relationships across populations and taxa.

### 2.2 Human dimension

Urban organisms are relatively understudied, in part because of a historical perspective that urban populations provided little value in understanding “natural” selection due to their proximity to humans, or because of their perceived lack of potential for novel research (McPhearson et al., 2016; Sukopp, 1998). Evolutionary ecology has only experienced a recent, but growing, appreciation of urban ecosystems (Rivkin et al., 2019; Szulkin, Garroway, et al., 2020), relying on decades of natural history research in non‐urban systems to develop hypotheses of urban adaptation. Importantly, the human biases in organism focus, specimen collection, and deposition into museum collections have resulted in a paucity of historical resources for many urban organisms, making urban retrospective analyses more difficult, particularly for human commensal species (Shultz et al., 2020; but see Major & Parsons, 2010; Meineke & Davies, 2019). Although many environments and taxa have been historically understudied, urban environments and their associated organisms have been systemically understudied until recently. Consequently, relatively little urban historical data exists across taxonomic and geographic extents compared to other globally distributed habitats (e.g., tropical rainforests) or global non‐urban taxa (e.g., stickleback fish). Framing contemporary adaptations in a historical context is important because species may evolve through novel adaptations in the contemporary and changing selective landscape or through filtering, or adaptive modification of existing traits (i.e., exaptations; Gould & Vrba, 1982; McDonnell & Hahs, 2015; Rivkin et al., 2019).

### 2.3 Misconceptions

A misconception perpetuated by knowledge gaps in the natural history of urban organisms is that non‐urban or historic populations are always appropriate baselines in a comparative framework. Although such comparisons are often informative, if we do not know how trait–environment relationships differ within and between urban environments then we may be misled about the nature of adaptation by employing an inappropriate baseline. Inaccurate inference of present interactions between traits and urbanization hinders our ability to make informed predictions about urban adaptation. These gaps in natural history knowledge are particularly consequential for species that are more common in urban areas than in non‐urban areas, such as rats and pigeons. For example, urban rats (*Rattus norvegicus* and *R. rattus*) have been placed in historical contexts mainly from archeological collections because museum collections lack specimens of the species that commonly cohabitate with humans (Guiry & Buckley, 2018), and so we might not know the true ancestral state to urban adaptive responses. In extreme circumstances where we have no contrast at all with non‐urban populations, such as with the common bedbug (*Cimex lectularius*), we might reach incorrect conclusions about how they have adapted specifically to urban environments based solely on their present adapted state (Gould & Lewontin, 1979).

### 2.4 Moving forward

To address gaps in knowledge regarding the natural history and trait–environment relationships in urban organisms, integrated research that combines observational studies (e.g., natural history and behavioral research) with experimental data of species living in cities is important. One approach to building a foundation of natural history information for urban organisms that have been successful in non‐urban environments (Fontaine et al., 2021; Sforzi et al., 2018) is to incorporate community‐sourced data collection into research. For example, Puckett et al. (2020) used museum specimens to study changes in brown rat cranial shape over time, and Cosentino and Gibbs (2022) used community‐sourced data to demonstrate the parallel evolution of clines in melanic Eastern gray squirrels (*Sciurus carolinensis*). However, community‐sourced data is often limited as a result of socioeconomic biases of regions sampled or as a result of limited sampling of overlooked, camouflaged, or microscopic species that are less charismatic (Shirey et al., 2021). Although we cannot go back in time to improve historical collections, moving forward we can deliberately start prioritizing collecting the types of data in urban ecosystems that we will need in future, including museum specimens (Shultz et al., 2020). Community partnerships in overlooked geographic regions can provide a more comprehensive sampling of the urban landscape (Shirey et al., 2021), while also augmenting museum collections with urban organisms and building stronger relationships with local communities. Moreover, equitable community partnerships provide benefits to both visiting scientists and local communities, facilitate access to research products, reduce the potential for conflict, and provide valuable outreach opportunities (Haelewaters et al., 2021; Sforzi et al., 2018; Shirey et al., 2021; Shultz et al., 2020).

## 3 CHALLENGE 3: AGENTS AND TARGETS OF SELECTION

### 3.1 General application

Quantifying the agents and targets of natural selection is essential for understanding local adaptation (Kawecki & Ebert, 2004) in any environment, yet is inherently difficult (Endler, 1986). Targets of selection may be misidentified or confounded in both phenotypic and genomic approaches due to a poor understanding of the relationships between genotype, phenotype, and environment (Bierne et al., 2011; Hoban et al., 2016; Linnen & Hoekstra, 2009). Disentangling selection on single versus multiple correlated traits can be particularly difficult because of genetic, developmental, and functional constraints (Hill & Robertson, 1966; Lande & Arnold, 1983; Price, 1970). The genetic architecture of a phenotype can also complicate genomic tests for local adaptation as selection on polygenic traits may be more difficult to detect in genomic scans compared to single locus traits (Hoban et al., 2016). Given the suspected prevalence and importance of polygenic adaptation and that rapid adaptation may involve soft rather than hard selective sweeps, identifying genomic targets of selection may be difficult for many complex phenotypes (Messer & Petrov, 2013; Rockman, 2011). In addition, large sample sizes and complex statistical methods may be required to detect changes in genotype in what are typically small selection coefficients (Kingsolver et al., 2001), and episodic or age‐specific selection may not be discernable as to when selection has occurred (Grant & Grant, 2014). The signatures of past and contemporary selection can be difficult to differentiate (Haller & Hendry, 2014) as phenotypes may arise in response to selective pressures in the contemporary environment but also may have arisen under ancestral selective regimes (i.e., are exaptations) or as a consequence of nonadaptive processes (e.g., gene flow). Lastly, in any environment humans can directly or indirectly change factors affecting selection and adaptation such as resource availability, resource distribution, population connectivity, and habitat size.

### 3.2 Human dimension

The urban environment is human‐built, thus many of the agents of selection are anthropogenic and not previously encountered by organisms or researchers in non‐urban environments (Alberti, 2015; Lugo et al., 2018). For example, extensive impervious surfaces (e.g., asphalt) within cities can impact local climate because they absorb and radiate solar energy differently than natural substrates (the “urban heat island” effect, Oke, 1973), and high concentrations of anthropogenic pollutants in urban habitats could accelerate mutation rates (Johnson & Munshi‐South, 2017; Somers et al., 2004; Yauk et al., 2000). Understanding these anthropogenic pressures may require cross‐disciplinary collaboration (e.g., engineering, physics, chemistry, governance, and urban planning; McPhearson et al., 2016). Moreover, teasing apart the relative importance of local adaptation, exaptation, and nonadaptive (e.g., gene flow) origins of urban phenotypes can be particularly challenging in urban environments. For example, as a consequence of human‐associated population connectivity, pigeons (*Columba livia*) in the Northeastern United States form a large continuous genetic metapopulation spanning city centers separated by over 800 km (Carlen & Munshi‐South, 2020). In fact, due to human‐mediated movement, some organisms have a higher probability, frequency, and distance of dispersal in somewhat predictable ways (e.g., intercity translocations; Bennett et al., 2019; Gotzek et al., 2015). For example, urban areas act as hubs to increase connectivity among populations of the Western black widow spider (*Latrodectus hesperus*), including among historically and geographically distinct populations locally adapted to desert environments (Miles, Dyer, & Verrelli, 2018; Miles, Johnson, et al., 2018).

### 3.3 Misconceptions

A misconception perpetuated by poorly understood agents and targets of selection is that selection in urban environments is strong primarily as a consequence of humans and human activities as agents. Although rates of phenotypic change have been demonstrated to be elevated in response to some anthropogenic agents (Alberti, 2015; Hendry et al., 2008; Whitehead et al., 2012), many studies rely on environmental proxies such as impervious surface cover rather than identifying causal relationships. Researchers may conflate environmental proxies with drivers of selection if the selective agents are unclear, multicollinear, or correlated with general environmental features*—*a problem that plagues adaptation research in any environment (Endler, 1986; Kawecki & Ebert, 2004; Mitchell‐Olds & Shaw, 1987). For example, in urban crested anoles (*A. cristatellus*), limb length differences can be connected to shifts in the structural environment directly related to locomotion (Winchell et al., 2016, 2018), although this trait shift could also be explained by the proxy variable of impervious surface cover correlated with the structural environment. In addition, contemporary movement patterns of urban organisms influenced directly and indirectly by human activities can obscure the selective landscape that shaped phenotypes. For example, populations of the mosquito *Culex pipiens* were presumed to be locally adapted to living in subway stations in London, yet a recent review instead supports exaptive origins of these underground‐adapted populations, with adaptive phenotypes previously present in the ancestral populations outside of Europe (Haba & McBride, 2022). As in any environment, if we fail to first characterize patterns of gene flow and genetic drift, we may incorrectly conclude local adaptation to urban environments (e.g., Gould & Lewontin, 1979; Hoban et al., 2016).

### 3.4 Moving forward

To address the challenges of understanding novel anthropogenic selective pressures, connecting phenotypes to selective agents and accounting for nonadaptive processes is crucial (Miles et al., 2019; Santangelo et al., 2018). Research that connects adaptive urban phenotypes to selective agents through performance or fitness quantification (e.g., Chick et al., 2020; Tüzün & Stoks, 2020) will provide more informative evidence of urban adaptation and reduce the conflation of environmental proxies (e.g., general urban characteristics) with drivers of phenotypic change. Genomic approaches may be particularly valuable to examine adaptive responses while accounting for underlying population structure. For example, Salmón et al. (2021) used genotype‐environment association tests to identify adaptation in the great tit (*Parus major*) across multiple cities, interpreting results in light of population structure analyses suggesting widespread gene flow across city centers. When populations are highly connected, it can be unclear if adaptive phenotypes arose repeatedly or swept across urban populations, a subtle distinction in the evolutionary mechanism underlying adaptation. Teasing apart these mechanisms is possible: Oziolor et al. (2019) used a model developed by Lee and Coop (2019) to determine how both de novo mutation and adaptive introgression contributed to pollution tolerance in Gulf killifish (*Fundulus grandis*). Lastly, long‐term datasets, including building museum resources (see Challenge 2) and research on ancient DNA will provide an important context for understanding urban adaptation by addressing temporal variation and timescales in natural selection. For example, in non‐urban ecosystems, the selection of beak size in Galapagos finches (*Geospiza* spp.) fluctuates from year to year in variable directions, and by building a multidecadal data set, Grant and Grant (2014) were able to quantify these dynamics.

## 4 CHALLENGE 4: HABITAT HETEROGENEITY

### 4.1 General application

The scale at which adaptation research is conducted must consider the breadth of habitats in an environment (Castillo & De León, 2021; Levin, 1992), across which the strength and nature of selection may vary. Qualitative habitat categorizations (e.g., montane and lowland) may not capture the habitat features underlying selection and adaptation, particularly at organismally relevant (e.g., microhabitat) spatial scales (Castillo & De León, 2021). Quantifying habitat at local spatial scales is important because similar habitat use (e.g., thermal niche) can impede adaptive divergence between populations occupying divergent macrohabitats (e.g., cool montane versus warm lowland; Muñoz & Losos, 2018). In addition, quantifying the extent of environmental divergence across habitat contrasts establishes the premise that similar selective forces underlie the covariation between phenotype and fitness, without which the selective landscape may be oversimplified, and proxies (e.g., macrohabitat elements) may erroneously appear to be the main drivers of selection (see Challenge 3). For example, macroclimatic variables (e.g., temperature and precipitation) were weak predictors of niche evolution in plethodontid salamanders in contrast to microhabitat variables (e.g., air temperature, soil temperature, leaf litter depth; Farallo et al., 2020). In addition to spatial variation, all habitats change over time as a consequence of natural processes (e.g., hurricanes, succession) as well as human activity (e.g., land management tied to social and political priorities; Ian Perry & Ommer, 2003). Adaptation research that considers temporal variation in the selective landscape may help with minimizing disruption of experiments (e.g., increased community collaboration, see Challenge 1) and identifying appropriate temporal windows of selection (e.g., better understanding of when selection is operating, see Challenge 3).

### 4.2 Human dimension

Modern urbanization represents a significant shift in the complexity, speed, and scope of human modification of the environment on both temporal and spatial scales (United Nations Center for Human Settlement (HABITAT), 2001). Examples of anthropogenic habitat transformation include expansion or contraction of infrastructure, landscaping, and extreme disturbances that radically and rapidly obliterate entire metropolitan areas (such as the recent war conflict in Ukraine). Anthropogenic environmental transformations have long‐lasting effects on evolutionary processes in urban environments by altering habitat characteristics and connectivity (Des Roches et al., 2021; Pincetl, 2015; Schell et al., 2020). For example, railways in German cities facilitated movement in admixed lineages of wall lizards (*Podarcis muralis*) derived from populations in other European cities (Beninde et al., 2018). In addition, socio‐cultural aspects of urban environments, including the legacy of urban development and discriminatory practices that promote structural racism (e.g., restrictive and discriminatory property sales), generate a heterogeneous landscape and idiosyncratic variation within and between urban centers (Des Roches et al., 2021; Pincetl, 2015; Schell et al., 2020; United Nations Department of Economic and Social Affairs & Population Division, 2018; Yigitcanlar, 2009). For example, wealthy communities often have more green space with abundant domesticated and invasive vegetation compared to poorer communities (Aronson et al., 2014). In addition, modern urbanization in North and South America is more recent than in Asia and Europe (Fox & Goodfellow, 2016), leading to less time for urban adaptation to have occurred in American cities. It might be the case that given the relatively recent age of most cities on Earth (a large proportion of which emerged or radically expanded after the Industrial Revolution and are less than 200 years old), adaptation may occur primarily from standing genetic variation rather than de novo mutation and result in primarily soft sweeps that are more difficult to detect using classic genomic approaches (Messer & Petrov, 2013). However, the importance of standing genetic variation for urban adaptation, and how this relates to variation among cities, remains understudied. Even in urban regions that have existed for centuries, human interests and needs (e.g., roads and energy infrastructure) can lead to drastically different selective landscapes at different points in time. For example, Paris was radically transformed in the 19th century by demolishing overcrowded medieval neighborhoods and building new parks and squares (Kirkland, 2013) indicating that urban landscapes continue to dynamically change over time.

### 4.3 Misconceptions

A misconception perpetuated by our nascent understanding of the heterogeneity of cities is that urban environments represent replicated natural experiments with parallel environmental conditions and selective pressures within as well as across cities globally (Diamond & Martin, 2021; Santangelo et al., 2022; Santangelo, Miles, et al., 2020; Santangelo, Thompson, et al., 2020; Szulkin, Garroway, et al., 2020). Although accumulating evidence suggests urban environments do converge on multiple environmental variables (e.g., Santangelo et al., 2022), the majority of urban adaptation research to date focuses on single geographic and established study regions (Santangelo, Miles, et al., 2020). However, we now recognize that replication within a single city, as well as contrasts of urban versus non‐urban habitats or across urban to non‐urban gradients, may ignore the complex mosaic of anthropogenically impacted landscapes that vary within and among cities (Szulkin, Garroway, et al., 2020). Although we have many operative definitions of “urban” environments, there is not a universal consensus on what defines a city. For example, variation in biotic (e.g., ecological dynamics), abiotic (e.g., temperature), and social factors (e.g., political structures) within and across urban environments may be underappreciated because of the North American and Western European focus of much of urban evolutionary ecology research (Des Roches et al., 2021; Johnson & Munshi‐South, 2017; Schell et al., 2020). Therefore, we may reach incorrect conclusions about the generalizability of urban adaptations globally based on this biased sample of urbanization.

### 4.4 Moving forward

To address the challenges presented by the inherent heterogeneity within and among urban environments, it could benefit researchers to move past a simplified assumption of cities as replicates to incorporate heterogeneity and scale more explicitly. Accomplishing this might involve quantification of urbanization at multiple spatial scales and replication across diverse cities globally (Pincetl, 2015; Szulkin, Garroway, et al., 2020). For example, Merckx et al. (2018) employed spatially hierarchical sampling to capture the regional and local variation of temperature and fragmentation in three city centers to understand adaptive patterns of invertebrate body size. When assessing multiple spatial scales is not feasible (e.g., remote‐sensing data of appropriate resolution is unavailable or access to field locations is restricted), a biologically‐justified scale that reflects local organismal interactions with their environment (e.g., dispersal or home range) can be used as a proxy (Jackson & Fahrig, 2015; Szulkin, Garroway, et al., 2020). Critically, such decisions rely on natural history and trait–environment information that may not yet be available for urban organisms (see Challenge 2), and different methods may be more appropriate (e.g., depending on spatial and temporal variation), requiring flexibility in experimental designs and interdisciplinary collaborations (see Challenge 1). In addition to a more quantitative assessment of urban environments, the global study of cities that vary in the intensity, age, and characteristics of urbanization will help shed light on the process of urban adaptation and aid in our ability to generalize findings. For example, Cosentino and Gibbs (2022) were able to disentangle selective agents contributing to parallel and non‐parallel clines in Eastern Gray Squirrel (*S. carolinensis*) melanic coat color associated with urbanization by comparing 43 North American cities that differed in size, age, and geographic location. In a global sample, Santangelo et al. (2022) collected data on white clover (*Trifolium repens*) from over 160 cities worldwide to demonstrate that urbanization can lead to parallel adaptation despite considerable environmental variation among cities.

## 5 CLOSING REMARKS

**FIGURE 2:**
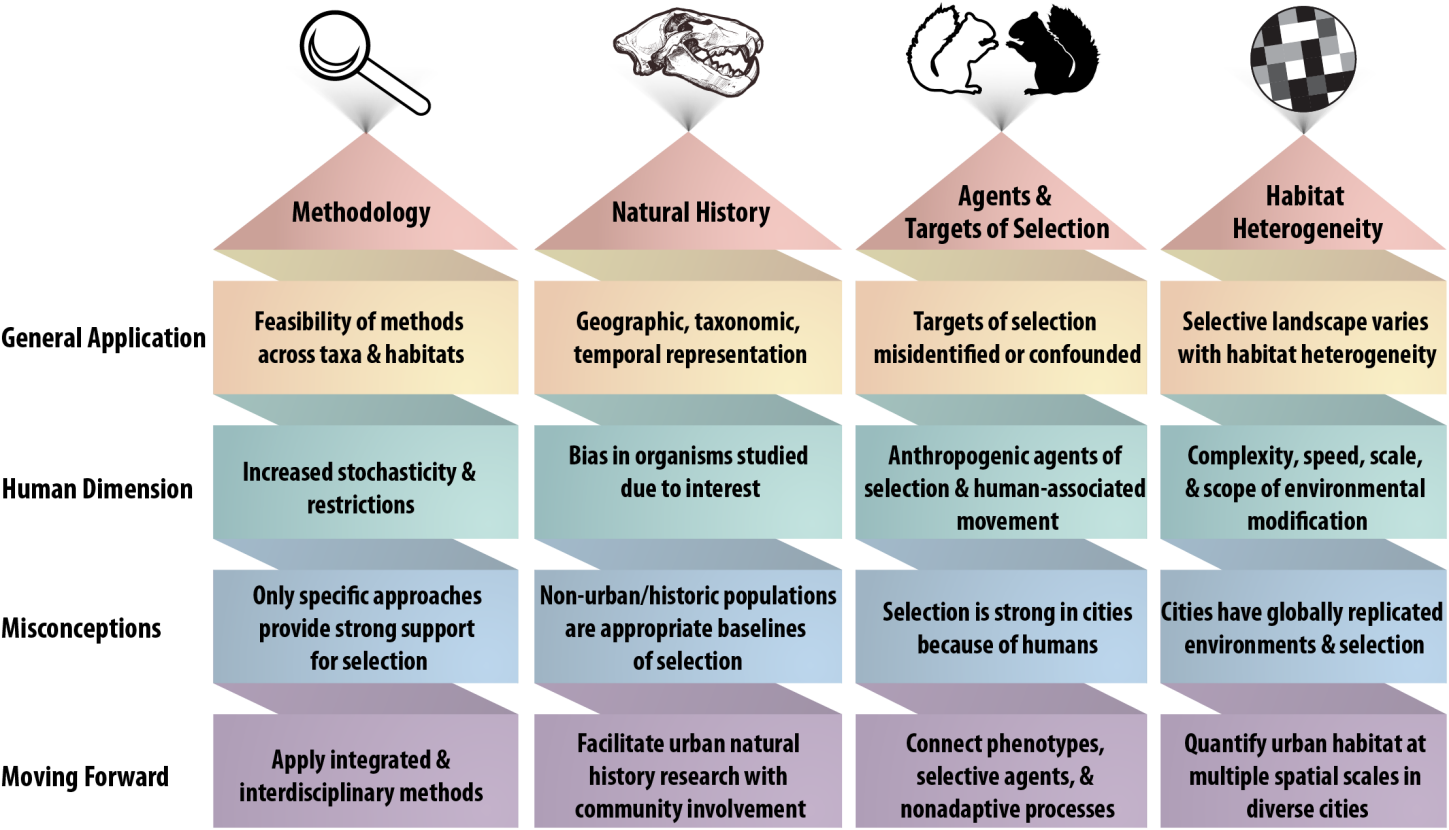
Urban adaptation research may be challenging as a consequence of increased interactions with and influence of humans in urban environments. We discuss challenges and ways to move forward from these main themes: Methodology, natural history, agents and targets of selection, and habitat heterogeneity.

Here, we have addressed some of the challenges researchers face when embarking on adaptation research in urban environments related to four themes: methodological approaches, trait–environment relationships and natural history, agents and targets of natural selection, and habitat heterogeneity (Figure 2). Although these challenges are not unique to urban environments, there are unique aspects that stem from the human dimension in these ecosystems. When researchers study urban evolutionary processes without considering these challenges, erroneous conclusions can arise regarding the nature and strength of selection, as well as the generalizability of findings across taxa and cities. Developing an understanding and appreciation of the human dimension and how it challenges adaptation research has broad applications to the diverse socio‐cultural aspects of urban ecosystems, including the evolution of urban organisms. As government and other agencies align their funding roadmaps with urban research, we believe outlining these challenges from biological, methodological, theoretical, and socio‐cultural perspectives is critical to the success of the field.

Although we have focused on the challenges of conducting urban adaptation research, we also recognize that urban areas are rapidly evolving environments that are globally distributed, and thus are powerful opportunities for contemporary adaptation research (Diamond & Martin, 2021; Donihue & Lambert, 2015; Szulkin, Munshi‐South, & Charmantier, 2020). This is not to say that urban ecosystems are qualitatively “good,” nor are they more appropriate than non‐urban systems for adaptation research. In fact, how humans interact with and influence nature cannot be extricated from wildlife conservation practices (Bergey & Whipkey, 2020; Egerer & Buchholz, 2021; McKinney, 2006). Even so, cities provide the opportunity to study ecological interactions and evolutionary outcomes that may uniquely result from the dynamic interactions that include humans. In addition, adaptation research can utilize aspects of urban ecosystems to carry out research that would otherwise be challenging or not possible in non‐urban systems. For example, habitat fragmentation and the frequent and ongoing management actions in cities can be leveraged to test hypotheses about connectivity without needing to actively modify the habitat. Indirect consequences of human activities also offer natural “laboratories” for addressing some of the most pressing issues of the Anthropocene. For example, cities can be viewed as experimental arenas to study adaptation to climate change because of the urban heat island effect (Oke, 1973), which in some ways is a spatial analogy of climate change (a temporal trend, Verheyen et al., 2019), and allows for a broader perspective on adaptation to warming than would be possible with laboratory experiments (Lahr et al., 2018). Similarly, cities increase the scope for the study of adaptation to anthropogenic materials such as plastic or other solid waste found in the environment, for example when these are used as replacement materials in biological structures. This can be best illustrated in nest building, viewed as an extended phenotype, when natural nest‐building elements such as fur and feathers are replaced by anthropogenic solid waste pollutants such as plastic or paper (Jagiello et al., 2022).

Finally, the generation and application of ecological and evolutionary information in urban areas may be facilitated because these ecosystems are intimately integrated with human societies. For some types of data such as historical land use and aerial imagery, researchers may find more resources for urban areas than non‐urban areas, although there may be geographic biases in the quality and temporal extent of these resources. Urban environments also provide an opportunity to learn about the ecosystems where we live and within which we have a vested interest. Community applications follow naturally from urban research via: regular interactions with the public while conducting fieldwork; museum exhibitions highlighting urban ecosystems (e.g., Carnegie Museum of Natural History's 2017 “We are Nature” exhibit); community science initiatives that involve urban communities in research activities (e.g., iNaturalist, BioBlitzes, SquirrelMapper: Cosentino & Gibbs, 2022) and interdisciplinary projects in urban spaces involving policymakers, artists, educators, and researchers (Sexton et al., 2015; Vega et al., 2021; Wallis et al., 2021). By conducting research on how the organisms around us are adapting to human modifications of the environment, we celebrate the diversity of where we live and engage communities to discover and celebrate this diversity. Ultimately, these initiatives expose those who live within cities to the excitement of evolutionary ecology and foster a sense of environmental stewardship.

## AUTHOR CONTRIBUTIONS

**Kristin Winchell:** Conceptualization (equal); project administration (lead); writing – original draft (equal); writing – review and editing (equal). **Kevin J. Aviles‐Rodriguez:** Conceptualization (equal); project administration (supporting); writing – original draft (equal); writing – review and editing (equal). **Elizabeth J. Carlen:** Conceptualization (equal); project administration (supporting); writing – original draft (equal); writing – review and editing (equal). **Lindsay S. Miles:** Conceptualization (equal); project administration (supporting); writing – original draft (equal); writing – review and editing (equal). **Anne Charmantier:** Conceptualization (supporting); writing – original draft (equal); writing – review and editing (equal). **Luis F. De León:** Conceptualization (supporting); writing – original draft (equal); writing – review and editing (equal). **Kiyoko Gotanda:** Conceptualization (supporting); writing – original draft (equal); writing – review and editing (equal). **L. Ruth Rivkin:** Conceptualization (supporting); writing – original draft (equal); writing – review and editing (equal). **Marta Szulkin:** Conceptualization (supporting); writing – original draft (equal); writing – review and editing (equal). **Brian C. Verrelli:** Conceptualization (equal); project administration (lead); writing – original draft (equal); writing – review and editing (equal).

## Data Availability

There are no data associated with this manuscript.
